# Ethical Conundrums in the Application of Artificial Intelligence (AI) in Healthcare—A Scoping Review of Reviews

**DOI:** 10.3390/jpm12111914

**Published:** 2022-11-16

**Authors:** Sreenidhi Prakash, Jyotsna Needamangalam Balaji, Ashish Joshi, Krishna Mohan Surapaneni

**Affiliations:** 1 Panimalar Medical College Hospital & Research Institute, Varadharajapuram, Poonamallee, Chennai 600 123, Tamil Nadu, India; 2 School of Public Health, The University of Memphis, Memphis, TN 38152, USA; 3 SMAART Population Health Informatics Intervention Center, Foundation of Healthcare Technologies Society, Panimalar Medical College Hospital & Research Institute, Varadharajapuram, Poonamallee, Chennai 600 123, Tamil Nadu, India; 4 Bioethics Unit, Panimalar Medical College Hospital & Research Institute, Varadharajapuram, Poonamallee, Chennai 600 123, Tamil Nadu, India; 5 Departments of Biochemistry, Medical Education, Molecular Virology, Research, Clinical Skills & Simulation, Panimalar Medical College Hospital & Research Institute, Varadharajapuram, Poonamallee, Chennai 600 123, Tamil Nadu, India

**Keywords:** artificial intelligence, machine learning, deep learning, ethics, medical ethics, ethical complications, autonomy, artificial intelligence in healthcare, legal and ethical guidelines, application in healthcare

## Abstract

Background: With the availability of extensive health data, artificial intelligence has an inordinate capability to expedite medical explorations and revamp healthcare.Artificial intelligence is set to reform the practice of medicine soon. Despite the mammoth advantages of artificial intelligence in the medical field, there exists inconsistency in the ethical and legal framework for the application of AI in healthcare. Although research has been conducted by various medical disciplines investigating the ethical implications of artificial intelligence in the healthcare setting, the literature lacks a holistic approach. Objective: The purpose of this review is to ascertain the ethical concerns of AI applications in healthcare, to identify the knowledge gaps and provide recommendations for an ethical and legal framework. Methodology: Electronic databases Pub Med and Google Scholar were extensively searched based on the search strategy pertaining to the purpose of this review. Further screening of the included articles was done on the grounds of the inclusion and exclusion criteria. Results: The search yielded a total of 1238 articles, out of which 16 articles were identified to be eligible for this review. The selection was strictly based on the inclusion and exclusion criteria mentioned in the manuscript. Conclusion: Artificial intelligence (AI) is an exceedingly puissant technology, with the prospect of advancing medical practice in the years to come. Nevertheless, AI brings with it a colossally abundant number of ethical and legal problems associated with its application in healthcare. There are manifold stakeholders in the legal and ethical issues revolving around AI and medicine. Thus, a multifaceted approach involving policymakers, developers, healthcare providers and patients is crucial to arrive at a feasible solution for mitigating the legal and ethical problems pertaining to AI in healthcare.

## 1. Introduction

### 1.1. Background and Rationale

Artificial Intelligence (AI) can be defined as “the part of digital technology that indicates the utility of coded computer technology routines with specific instructions to carry out tasks for which generally a human brain is considered necessary” [1]. The emergence of artificial intelligence is one of the most profound advancements in the history of medicine, with applications in all the medical specialties [2]. Artificial intelligence bears stupendous promise in terms of propitious, precise and efficacious preventive treatment and curative interventions [3]. The principal class of applications includes diagnosis and treatment strategies, patient adherence to drug regimens and hospital administrative affairs [4]. AI applications in hospitals are instrumental in alleviating the pressure on healthcare professionals, are cost-effective and, in the long run, improve the quality of healthcare services [5]. AI can be deployed for structuring the existing medical data by transforming colloquial language into a clinical narrative, scrutinizing patient data, recognizing clinical diagnostic resemblances, as well as substantiating medical hypotheses [6]. The application of artificial intelligence in healthcare delivery, nonetheless, gives rise to transnational ethical, legal, social and commercial concerns. The utilization of digital technologies and software in healthcare facilities has created testing circumstances for software developers, policymakers and healthcare providers. AI presents ethical concerns that impede the progress of its usage in the healthcare field [7].

The use of AI in healthcare takes advantage of the exceptional volume of health data and computing potential to inform evidence-based decision-making. This paves the way for new ethical dilemmas pertaining to the confidentiality of the data collected, data privacy, the transparency of data use, accountability of data governance and probable inequities in AI deployment [8]. Most of the algorithms of artificial intelligence function inside a “black box” environment, where the course of analysis is not transparent. AI is subject to basic ethical complications related to autonomy, beneficence, justice, non-maleficence and respect for knowledge. In the healthcare system, the privacy of an individual should be revered, as is obliged by patient autonomy or self-governance, personal identity and well-being. Henceforth, it is ethically vital to give due respect to patients’ privacy and maintain confidentiality [9]. Artificial intelligence and machine learning systems have an algorithmic bias, predicting the probable diagnosis of a disease on the grounds of sex or race, when these might not be the key causative factors [4]. One feature that differentiates the medical industry from other service sectors is the unconditional trust patients have in healthcare professionals, which is reinforced by the placebo effect. In AI-assisted healthcare, patients have to construct a relationship with an artificial system rather than a human. This significantly affects the treatment outcomes [7].

Technological and scientific advancements and the ubiquitous presence of digital technology have facilitated the establishment of a unified trust in digital space. The main purpose of modern digital legal regulation encompassing AI technology in medical care is, fundamentally, to impede the emergence of public health risks and give due respect to the confidentiality of patients’ private data. In February 2017, European legislation approved the use of AI technology in healthcare under the conditions that a robot should not cause harm to the users, should abide by human commands and ensure its safety [10]. The proposed act by the European Commission mandates the documentation of AI technology, data sheets with information on training modality and the process of implementation with their scope and characteristics [11]. In India, there are no concrete laws pertaining to AI in healthcare. DISHA, or the Digital Information Security in Healthcare Act, if brought into action, will cover the regulations under this domain [12].

The year 2015 marked the adoption of the Sustainable Development Goals by all the member states of the United Nations. The Sustainable Development Goals aim to achieve good health and well-being [13]. They also aim to reduce inequalities. While the goals are well-grounded on the ethical principles of equality, equity, universal solidarity and a commitment to exclude no one, the evolution of artificial intelligence has the potential to further aggravate health inequities. With the fast-paced propagation of AI technologies in the medical field, it is exigent to recognize and address the ethical implications comprehensively, in view of the able benefits of AI, and alleviate its potential harms [14,15]. An extensive outlook on every plausible ethical problem associated with AI in healthcare facilities is required. To that end, our study aimed to perform a scoping review of the scholarly studies published in peer-reviewed journals to determine the ethical concerns with the application of AI in healthcare.

### 1.2. Objectives

This study aims to scrutinize the ethical complications associated with the application of artificial intelligence in the healthcare field. This scoping review further extends to put forth recommendations and guidelines for an ethical and legal framework.

## 2. Methodology

### 2.1. Search Strategy

The description of this scoping review is extracted from scholarly articles from the PubMed and Google Scholar databases describing the ethical issues related to AI in healthcare. A five-stage methodological framework from Arksey and O’Malley that included the following steps was used: 1. Defining the research topic. 2. Identifying relevant research papers. 3. Selecting the study. 4. Charting the data. 5. Collating, reporting, and summarizing the findings. The structure of the scoping review follows the PRISMA extension for scoping reviews (PRISMA-Scr) [16].

### 2.2. Identification of Relevant Studies

An extensive search of articles was conducted over a period of 4 days from 24 September 2022 to 27 September 2022. Key search terms such as, ‘artificial intelligence’; ‘machine learning’; ‘deep learning’; ‘ethics’; ‘medical ethics’; ‘ethical complications’; ‘autonomy’; ‘artificial intelligence in healthcare’; ‘legal and ethical guidelines’; and ‘application in healthcare’ were applied to extract the filters. The inclusion and exclusion criteria are as follows:

### 2.3. Inclusion Criteria

- Articles reporting on all the key search topics (AI, health, ethics);
- Studies dealing with ethics in the application of artificial intelligence exclusively in healthcare/medicine;
- Qualitative\quantitative\mixed-method studies, literature reviews published in indexed and peer-reviewed journals and grey literature;
- Articles published in the English language.

### 2.4. Exclusion Criteria

- Studies elucidating ethical challenges in the application of AI in disciplines other than medicine;
- Manuscripts written in languages other than English.

### 2.5. Selection of Sources of Evidence

**Figure 1:**
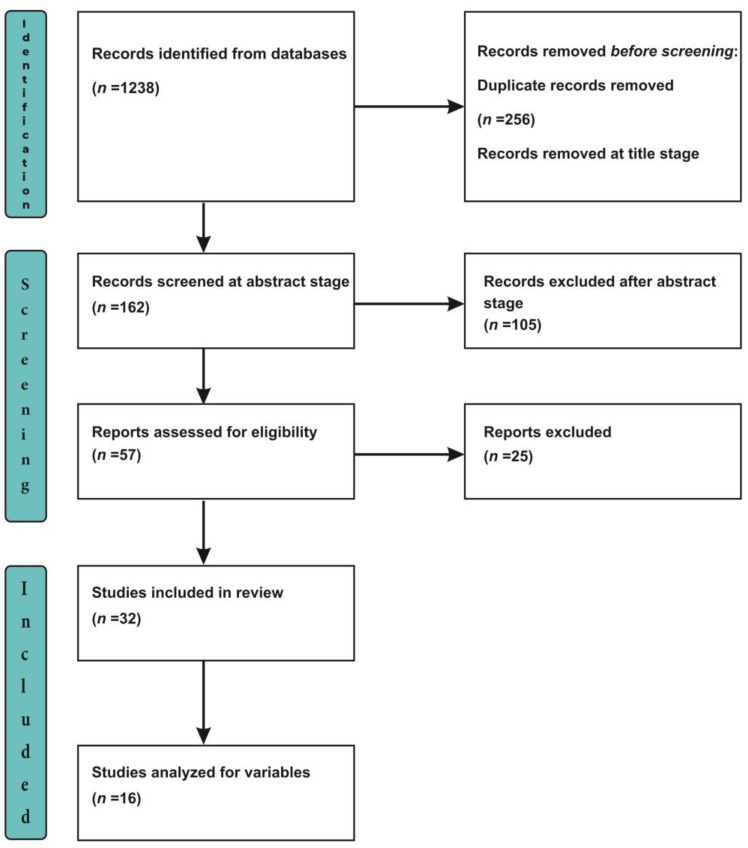
PRISMA Flowchart.

Using the Preferred Reporting Items for Systematic Reviews and Meta-Analysis (PRISMA) extension for Scoping Reviews (PRISMA-Scr) checklist [16], we extracted 1238 articles from our systematic search of the databases. A total of 256 articles were removed as they were identified to be duplicate records, and 820 articles were excluded at the title-screening stage as they were not relevant to the objectives of our present study. An abstract-stage screening was done for 162 articles, of which 57 articles were selected for further screening, with the exclusion of 105 articles at the abstract stage as they failed to meet the inclusion criteria for this review. In total, 32 articles were selected for the full-text screening, with the exclusion of 25 manuscripts (unable to locate full texts). Finally, 16 reviews were included in this scoping review, and 16 manuscripts were excluded (books\book chapters, comments, editorials, letters) as they did not fit into the inclusion criteria (Figure 1).

### 2.6. Data Charting

The data variables established from the articles analyzed for this study are the year of publication, type of review, aim, time frame of the search strategy, key words used to retrieve articles, key ethical issues discussed, major findings, conclusions and recommendations.

## 3. Results

### 3.1. Search Results

An extensive search of the PubMed and Google Scholar electronic databases yielded 1238 articles using appropriate keywords and, finally, 16 articles were selected for the present review on the grounds of the inclusion and exclusion criteria, along with the removal of duplicate articles (Figure 1).

### 3.2. List of Selected Studies

The records of studies analyzed for this scoping review are listed in Table 1, along with the study ID that will be used for further references in this manuscript.

### 3.3. Study Characteristics

The selected manuscripts were thoroughly analyzed and the following relevant variables were extracted for this scoping review (Table 2 and Table 3). An extensive data tabulation, which encompasses all the characteristic variables of the studies included, was sorted under the columns of the year of publication, study characteristics, aim, time period of study, keywords used for data retrieval, ethical issues discussed, major findings and conclusion and recommendations.

### 3.4. Analysis Study Characteristics

**Figure 2:**
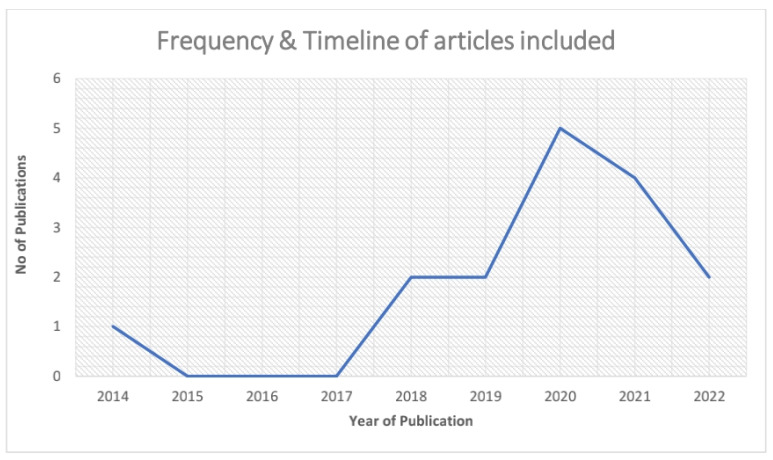
Depicts the Frequency & Timeline of articles that are included for this review.

The timeline of the studies included the earliest published article on the ethics of artificial intelligence in healthcare, which was in September 2014 [Study ID 1]. Five articles published in 2020, followed by four articles in 2021 and two articles each in 2018, 2019 and 2022 (Table 4 & Figure 2).

### 3.5. Keywords

**Figure 3:**
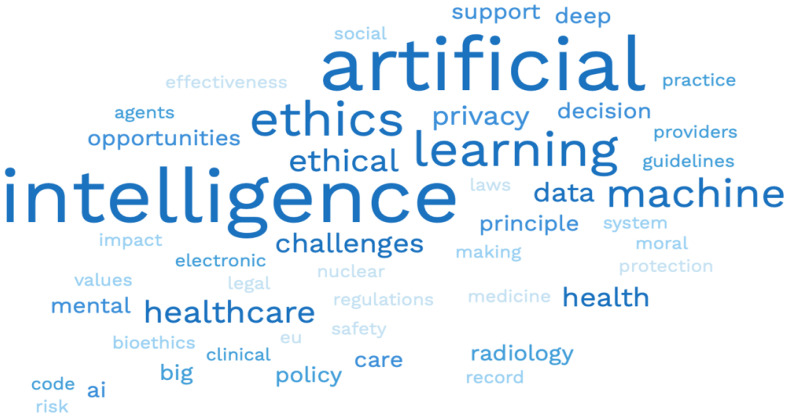
Displays the major key words used by the reviewers to retrieve the scientific literature for their studies.

The major keywords used by the reviewers to retrieve the scientific literature for their studies are illustrated in Figure 3. The most commonly used keyword is represented in darker tints of blue and less common words in lighter shades of blue.

### 3.6. Major Ethical Concerns

**Figure 4:**
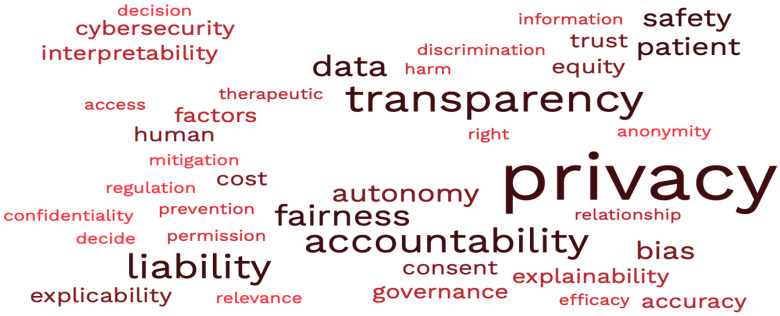
Illustrates the most frequently raised ethical issues regarding the application of artificial intelligence in healthcare.

The most frequently raised ethical issues regarding the application of artificial intelligence in healthcare settings are displayed in Figure 4. The predominant issues are highlighted in darker tints of red and the lesser addressed issues are displayed in lighter tints of red.

### 3.7. Synthesis of Results

Major ethical dilemmas and concerns over the implementation of artificial intelligence in healthcare and the potential solutions to the same have been outlined below:

(a) Predominantly, all the studies voiced concerns about the need to devise ethical principles and guidelines for facilitating the use of AI in healthcare; the existing code of laws and ethical frameworks are not up to date with the current or future application of artificial intelligence in healthcare. Considering the vulnerability of artificial intelligence to errors, patients prefer empathetic humans to treat them rather than artificial systems. However, an AI system, under the able supervision of healthcare professionals, has immense potential to bring about beneficial reforms in the healthcare system. In view of the massive scope for artificial intelligence in healthcare, it is obligatory for governments and other regulatory bodies to keep a check on the negative implications of AI in medical facilities.
(b) It was also found that the standard guidelines testing the applicability of AI upholding the ethical principles of fairness, justice, prevention of harm and autonomy are nonexistent. In spite of diverse research on artificial intelligence and its ethical implications, it was determined that the scientific data lacks a globally accepted ethical framework. The prevailing system of guidelines has been proven to be insufficient to assuage the ethical concerns about artificial systems in medicine. Wide scale revisions are needed in the current law and ethical codes to monitor AI in medical systems. Contrary to many reviews, a study conducted by Daniel Schonberger et al., in 2019, claimed that AI enhances the equity and equality of healthcare services, and the paucity of knowledge to sustainably implement AI is a major challenge in its application in healthcare [5].
(c) The ethical concerns ranging from data security to data privacy via the misuse of personal data have led to strained doctor–patient relationships. Achieving unimpeachable control over the risks associated with the use of AI plays a pivotal role. Concerns about using the obtained data, along with data protection and privacy, are important issues that need our attention for a successful artificial intelligence-driven healthcare administration. The optimum potential of AI in medical care cannot be achieved without addressing these ethical and legal conundrums. The meticulous planning of regulation and the implementation of AI is of utmost importance to harvest the maximum benefits from this unprecedented technology.
(d) The evolution of artificial intelligence and machine learning technologies has led to the development of a novel strategy called ‘co-design’. The concept of co-design has the ability to fix the loopholes in the existing code of ethics by actively involving stakeholders, software developers, policymakers, patients and healthcare professionals in ethical decision-making pertaining to artificial intelligence in healthcare. It is imperative for healthcare professionals to thoroughly study these innovative technologies to ethically implement them in clinical practice, which assists in mindful decision-making.

### 3.8. Recommendations Stated in the Articles Reviewed

Artificial intelligence systems can provide efficacious results in the healthcare system with the integration and standardization of the electronic health record regulatory system. A collaborative association comprising stakeholders, administrators and AI system developers can provide virtuous solutions to alleviate ethical hitches in the application of AI in healthcare. Moreover, a multifaceted, interdisciplinary approach actively involving the beneficiaries of AI in healthcare (patients) in the decision-making process in the implementation of AI in medicine is necessary. Data protection systems should be reinforced to prevent the seepage of patients’ personal data.

## 4. Discussion

The novel field of artificial intelligence is vigorous and growing at a fast pace. Even though the benefits of artificial intelligence in healthcare are versatile, its ethical concerns range from the diminishing nature of the doctor–patient relationship to the aggravation of existing health inequities [27]. The present review of scholarly articles on the ethics of artificial intelligence in healthcare aimed to explore the ethical complications associated with AI in healthcare and emphasize the ethical knowledge gaps in the literature by analyzing diverse studies conducted across the globe. The reviewed literature demonstrated an overwhelming ethical concern about data privacy, trust, accountability, bias, explicability, patient autonomy, fairness, transparency, data governance, confidentiality and data security [5,14,17,28,29]. These ethical drawbacks of AI applications in healthcare should be rigorously investigated and interpreted to utilize AI technologies to their maximum potential.

Autonomy is one of the four pillars of ethical principles in medicine. A qualitative study in the USA (conducted in 15 focus group discussions with 87 participants) reported that the participants, in general, have a welcoming and enthusiastic attitude towards AI in healthcare, as it has the potential to improve the care they receive at medical facilities, but they felt their patient safety, privacy and autonomy was at stake with the inclusion of artificial intelligence in healthcare. Patients should be given the freedom to decide if they wish to incorporate AI technology in their treatment care and be able to drop out of AI inclusion in their treatment at any point in the course of therapeutic treatment. The participants also raised concerns about the individuality of AI-assisted clinical decision-making, as they felt that every patient and clinical case scenario is unique [1,8,30]. Clinician–patient trust is built upon effective communication and relationships. They are crucial for the effectiveness of treatments and for improving treatment outcomes. In an AI-based healthcare delivery system, patients are confronted by artificial systems, and for those who have had hardly any exposure to digital technologies, establishing trust with an AI system can be arduous [7,31]. Privacy concerns over artificial intelligence in healthcare are ever-growing, with the circulation of confidential healthcare data across numerous unauthorized companies. This is inevitable, as machine learning and deep learning algorithms need an enormous amount of data in the process of training, testing and validating the algorithms [22,24,32,33].

AI-based decision-making lacks transparency. This feature of artificial intelligence, called the “black box” element, renders the AI decision-making process opaque [25,32]. Artificial intelligence should aid in achieving the well-being and safety of patients. Nevertheless, artificial intelligence has been proven to reinforce the existing biases in the healthcare industry [9,34]. Unreliable and under-representative data sets for AI development lead to inequity, data bias, discrimination and deceptive predictions. Health data are the most sensitive and intimate information. Respecting the privacy of the patient is a critical ethical principle, as privacy is built upon the grounds of autonomy and bound to individual identity and well-being [9,35].

These ethical considerations spotlight the significance of patient involvement to make sure that these novel technologies are incorporated into medical care in a way that strengthens patient trust and alleviates widespread ethical concerns about AI in healthcare. Furthermore, as patients are the target beneficiaries of all AI uses and innovations, characterizing their requirements, moral values and preferences is indispensable for ensuring that these novel technologies are developed, applied and implemented in an ethically appropriate way [8,36,37,38]. A multifaceted approach involving all the stakeholders, policymakers, patients and healthcare providers—in the application and implementation of AI in healthcare is needed [39].

AI has the capability to achieve universal health coverage, alleviate health and social inequities and enhance health outcomes on a larger scale. In view of the unclear boundaries of AI in health, we must be assiduous in responding to the ethical complications of its application in healthcare [40]. It is imperative to approach AI in healthcare with cautious enthusiasm, enlightened by a comprehensive body of ethical principles, to ensure its ethical application [14,41].

Artificial intelligence techniques can also be used for molecular analysis. Visible infrared spectroscopy data can be used as a significant quantitative indication for bimolecular investigations. This novel technique helps in obtaining spatial and spectral information as well as the spatial distribution of biomolecules to be analyzed. Moreover, the data transmission between the spectroscopy instruments and mobile devices is important for handheld field monitoring. Recently, it has been found that on-the-go communication technology is an effective mode of data transmission between spectroscopy equipment and mobile devices [42,43].

## 5. Knowledge Gaps

AI-driven healthcare practice has immense potential to add to the capacity and enrich the knowledge base of practicing clinicians. However, the existing literature clearly sets forth the ethical complications in the large-scale adoption of AI in healthcare. The literature largely lacks the findings from developing nations, as it is centered on developed countries. The most important component not addressed in the existing literature is a standard, universally acceptable framework or guidelines that ensure the ethical application of artificial intelligence in healthcare. The governance of artificial intelligence is of paramount importance, but the current literature review revealed a paucity of data on the governance of artificial intelligence in healthcare in different countries. Currently, very little research has been done characterizing the patients’ and other stakeholders’ perspectives on the application of AI in healthcare. Furthermore, the factors to be considered while preparing a checklist for AI ethics have not been explored. There is an acute paucity in the scientific research that elaborates on the ethical challenges and potential solutions to address the same. The lack of research on the ethical considerations, with only advancements in the technology, makes it difficult to implement and manage artificial intelligence interventions in the long run.

## 6. Directions for Future Research

There is a pressing demand for developing practical tools for testing the applicability of artificial intelligence in healthcare following ethical principles. Furthermore, future research is needed to recognize various stakeholders, users and beneficiaries of AI-related technology in medicine and to actively include them in dialogues about AI ethics and feasible solutions to establish ethical AI applications. For healthcare professionals to attain proficiency in artificial intelligence and implement it in daily clinical practice without compromising ethical principles, prior training is essential. To achieve this, it is imperative to incorporate artificial intelligence in undergraduate and postgraduate medical curricula, keeping in view the uniqueness of the various disciplines of medicine; extensive research investigating the ethical and legal problems of the AI application in diverse specialties and sub-specialties of healthcare is required. Comprehensive qualitative and quantitative studies, with an aim to decipher the perspectives of healthcare professionals and consumers of AI (patients), are needed.

## 7. Limitations

This study has certain inherent limitations. Due to the ever-expanding and evolving characteristics of artificial intelligence technologies, studies conducted since2014 were included in this review, as the findings of the studies before this time period may not be relevant to the recent developments seen in this digital era.

## 8. Conclusions

It is certain that AI will have extensive ramifications that transform healthcare practices, altering the patient experience and clinicians’ approach towards patients. However, a naïve implementation of artificial intelligence in healthcare may result in a wide array of ethical complications. There is an increasing demand for feasible solutions to the ethical implementations of AI in healthcare. This scoping review provided insights on patient privacy and autonomy, informed consent, transparency, fairness, data bias, inequity, the “black box” element, etc. There is a need to expand and codify the existing framework and organize it into categories connected to ethical principles such as autonomy, privacy, transparency, fairness and justice. It is crucial for healthcare facilities, governmental and regulatory organizations to build guidelines to tackle ethical issues, act in an accountable and responsible way and construct governance techniques to monitor the complications. Artificial intelligence is projected to have a high potential to transform healthcare. Universal standard guidelines for ethical and legal implications can lead to optimal utilization of AI in healthcare. We strongly believe that the establishment of a comprehensive and systematic ethical framework for AI application in healthcare can offer promising results in health outcomes.

## 9. Conceptual Framework

From the extensive review of the existing scientific literature, it is well established that there is an exigent need for an ethical framework for developing, implementing, governing and monitoring artificial intelligence in healthcare.

**Figure 5:**
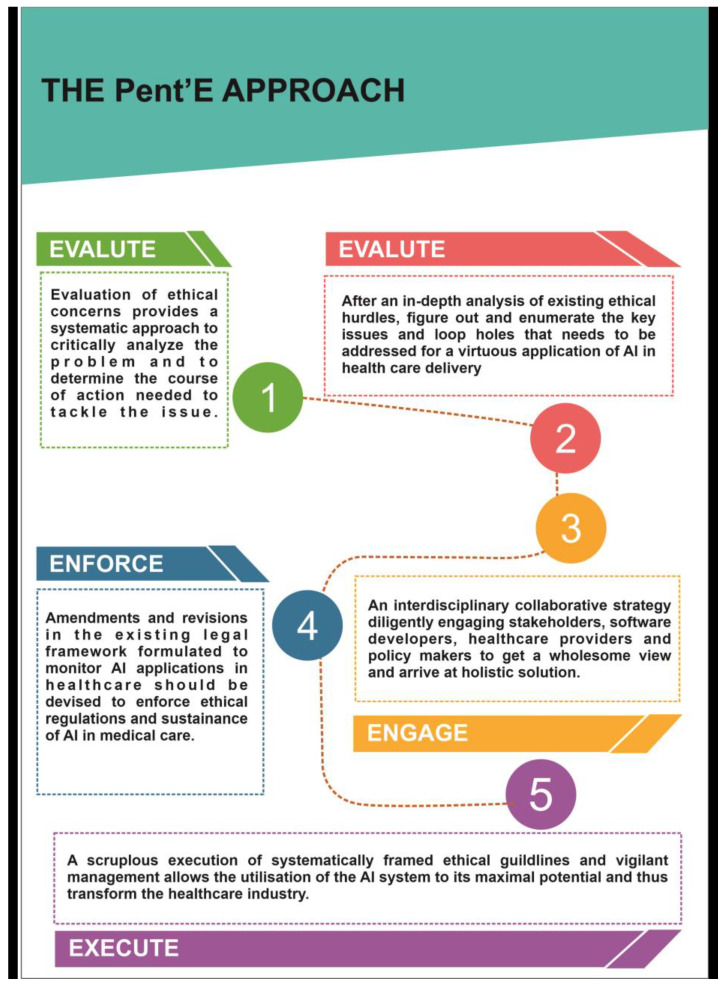
Enumerates the Pent’E approach of resolving ethical challenges in the application of AI in healthcare.

In this regard, we conceptualized the “Pent’E” approach that encompasses a five-step process of identifying, analyzing and implementing interventions to address the ethical and legal disputes in the application of artificial intelligence in healthcare for the benefit of patients (Figure 5).

The Pent’E approach:

1. EVALUATE
2. ENUMERATE
3. ENGAGE
4. ENFORCE
5. EXECUTE

The proposed framework consists of five main steps that range from problem identification to implementation. Critically analyzing the ethical challenges in the application of artificial intelligence in healthcare and appraising the existing literature for loopholes in the ethical and legal concerns will help in conceptualizing a plan to resolve these issues. This process will help in considering the current pressing issues in the ethics of artificial intelligence, and to further engage with stakeholders, policymakers, governing bodies, software developers and healthcare providers—to design, develop and deliver an efficient and ethically bound technical invention, with the enforcement of the current legal and ethical principles in favor of patients and healthcare providers, which strictly adheres to the principles of beneficence, non-maleficence, justice and autonomy. The final step is the execution of the planned course of action in a time-framed and effective manner, which brings to light all the ethical regulations and ways in a systematic way to widen the acceptance, utility and benefits of artificial intelligence in healthcare without any kind of malpractice.

## Figures and Tables

**Table 1:** Depicts the titles and first authors along with the study ID of the articles reviewed.

Study ID	Title	First Author
1	Recommendations for the ethical use and design of artificial intelligence care providers [17]	David D Luxton
2	The potential of artificial intelligence in healthcare [4]	Thomas Davenport
3	The ethical issues of the applications of artificial intelligence in healthcare: a systematic scoping review [18]	Golnar Karimian
4	Artificial intelligence in healthcare: a critical analysis of the legal and ethical implications [5]	Daniel Schonberger
5	Artificial intelligence in healthcare: opportunities and risk for future [9]	Sri Sunarti
6	Societal issues concerning the application of artificial intelligence in medicine [19]	Vellido A.
7	Ethical principles for the application of artificial intelligence in nuclear medicine [20]	Geoff Currie
8	How to achieve trustworthy artificial intelligence for health [1]	Kristine Baeroe
9	Ethical and legal challenges of artificial intelligence-driven healthcare [21]	Sara Gerke
10	Applications of artificial intelligence-based technologies in the healthcare industry: Opportunities and Challenges [7]	DonHee Lee
11	Balancing risks and benefits of artificial intelligence in the healthcare sector [3]	Kenneth Goodman
12	Artificial intelligence as a medical device in radiology: ethical and regulatory issues in Europe and the United States [22]	Filippo Pesapane
13	Ethics of artificial intelligence in medicine and ophthalmology [23]	Abdulla
14	Artificial intelligence in radiology: ethical considerations [24]	Adrian P. Brady
15	Identifying ethical considerations for machine learning healthcare applications [25]	Danton S, Char
16	iHealth: The ethics of artificial intelligence and big data in mental health care [26]	Giovanni Rubeis

**Table 2:** Characteristics of data extracted for analysis.

Study ID	Reference	Year of Publication	Study Characteristics	Aim	Time Period of Study	Key Words Used for Data Retrieval
1	[17]	2014	Review	To explore the ethical concerns related to AI in mental healthcare	Not mentioned	Artificial intelligence agents, ethics, ethical code, practice guidelines, care providers, mental health
2	[4]	2019	Review	To discuss on the ethical concern pertaining to application and implementation of AI healthcare	Not mentioned	Artificial intelligence, clinical decision support, electronic health record system
3	[18]	2022	Review	To determine the ethical problems of AI application in healthcare and enlist the knowledge gaps	Not mentioned	Artificial intelligence, machine learning, deep learning, ethics, bioethics
4	[5]	2019	Review	To give a wholesome view of ethical decision-making potential of AI	Not mentioned	Artificial intelligence, moral values, principle, decision making
5	[9]	2021	Review	To explore the risks posed by AI in healthcare	Papers published from 2010 to 2020	Artificial intelligence, healthcare, opportunities, risk
6	[19]	2018	Review	To reflect upon the aspects that affect the acceptance of AI in healthcare	Not mentioned	Artificial intelligence, machine learning, ethics, social impact, healthcare
7	[20]	2020	Review	To investigate the ethical principles of AI application in nuclear medicine	Not mentioned	Ethical principles, artificial intelligence, nuclear medicine, machine learning, deep learning
8	[1]	2020	Review	To discuss the ethical implications of AI technologies in healthcare	Not mentioned	Artificial intelligence, ethics, legal regulations, healthcare, machine learning.
9	[21]	2020	Review	To enumerate the trends and strategies in US and Europe forthe ethical implementation of AI in healthcare	Not mentioned	Artificial intelligence, ethical challenges, US and EU laws, safety and effectiveness, data protection and privacy
10	[7]	2021	Review	To study and analyze the global examples of AI in healthcare	Not mentioned	AI-based technology, real-world cases, opportunities and challenges, policy and management support, healthcare industry
11	[3]	2021	Review	To critically analyze the co-design model for mitigating the challenges of AI in healthcare	Not mentioned	Co-design, AI, ML, patient and public involvement, ethical implications, conceptual challenges
12	[22]	2018	Review	To analyze the ethical and regulatory concerns in using artificial intelligence for developing medical devices in radiology	Not mentioned	Artificial intelligence, legislation, policy, privacy, radiology
13	[23]	2021	Review	To explore the ethical concerns in the implementation of artificial intelligence in healthcare and drop them oncology and also to provide possible suggestions to create the ethical framework	October 2019 to April 2020	Artificial intelligence, ethics, oncology, cancer care.
14	[24]	2020	Review	To explore the ethical concerns in artificial intelligence in the field of radiology	Not mentioned	Artificial intelligence, radiology ethics, machine learning
15	[25]	2020	Review	To explore the potential challenges of the implication of artificial intelligence in pathology and laboratory medicine	Not mentioned	Ethics, artificial intelligence, machine learning, algorithm, privacy, big data
16	[26]	2022	Review	To analyze the ethics of artificial intelligence in the field of mental health care.	Not mentioned	Artificial intelligence, big data, ecological momentary assessment, ethics, mental health, self-motivation.

**Table 3:** Characteristics of data extracted for analysis.

Study ID	Reference	Ethical Issues Discussed	Major Findings	Conclusion	Recommendations
1	[17]	Therapeutic relationship, liability, trust, privacy and patient safety	Current code of ethics and guidelines does not take into consideration the present or future application of interactive artificial intelligence to aid or replace mental healthcare providers	The ethical principle regarding the use of AICP must be thoroughly devised for the use of the same in the future	Not mentioned
2	[4]	Accountability, transparency, permission and privacy	AI systems are vulnerable to errors. Patients may prefer empathetic clinician communication to medical principles received from robots	Considering the challenges of AI in healthcare it is vital for governmental regulators and healthcare bodies to monitor and limit adverse outcomes	For AI systems to be efficiently used in clinical practice certification from regulators HER system integration and standardization are important.
3	[18]	Human autonomy, prevention of harm, fairness, explicability and patient privacy	It was put forth that there exists no empirical guidelines or framework for checking the practicability of AI in upholding the ethical principle of fairness, prevention of harm, autonomy or explicability	AI technology is expanding at a rapid pace. Despite numerous research on the ethics of AI in healthcare, the literature lacks a universally accepted ethical guideline and framework	Interdisciplinary alliances between various stakeholders, legislative and administrative authorities and policymakers can corroborate the ethical application of AI in healthcare
4	[5]	Discrimination and liability	The existing framework of ethical guidelines is evidently insufficient to mitigate the ethical problems of AI in healthcare	Revisions are essential in the existing law and ethical frameworks	Not mentioned
5	[9]	Safety, efficacy, privacy, information and consent, cost, access, right to decide, equity, transparency, trust, accountability	The application of AI in healthcare will enhance prevention and treatment along with cost efficiency, equity and equality of healthcare services	The major challenges to AI in healthcare are the lack of sustainable implementation and no due respect to user viewpoints	Not mentioned
6	[19]	Explainability, interpretability, privacy, anonymity, fairness	Artificial intelligence in healthcare has immense beneficial outcomes provided the major public concerns over its usability, reliability, privacy and autonomy are evidentially resolved.	Collaboration between AI and ML developers and the medical community is required for formulating standard methods, protocols and guidelines for the ethical use of AI in healthcare	Not mentioned
7	[20]	Data governance, confidentiality, mitigation of bias, transparency, relevance, privacy, regulation, liability, accuracy, decision-making, acceptability, cost and equity	From data security to privacy, through misuse to shared accountability AI alters clinician patient relationship	AI though transformative, sets forth a number ethical and legal challenges that is worth our attention and needs formulation of guidelines	While creating and applying AI to healthcare, all the stakeholders, administrators, policy makers, healthcare workers and patients should be actively engaged
8	[1]	Respect for human autonomy, fairness, explicability, accountability, transparency, privacy and data governance	The existing frameworks are not directed towards addressing the obstacles to the achievement of trustworthy AI	Attaining trustworthy power over risks and harms related to AI is crucial	A globalized approach is needed to alleviate the potential harms of AI in healthcare
9	[21]	Informed consent to use, safety and transparency, algorithmic fairness and biases, data privacy, liability, cyber security, data protection and privacy	Informed consent, data protection and privacy and cyber security are all important factors that need to be considered for a virtuous AI-driven healthcare system	AI has the immense capability of enhancing healthcare systems, but its full potential can be utilized only by addressing ethical and legal challenges	Not mentioned
10	[7]	Accountability, AI divide, cyber security, privacy, loss of managerial control	Efficacious utilization of AI will need effective planning and implementation to reap the maximum benefits of the novel technology	Concerns over patient data prevails but no social principle has been developed on data ownership	Information protection systems should be made stronger to prevent the leak the patient data
11	[3]	Not applicable	AI\ML technologies have magnified the current challenges pertaining to patient and public involvement in ethical decision-making in healthcare	The concept of co-design has the potential to address the existing ethical and legal issues of AI in healthcare	Clarifying co-designs commitment to values; mapping socio-technical relations
12	[22]	Accountability	The application of AI in regulating the medical device best decision-making is highly unpredictable and therefore raises many ethical concerns.	Much research has to be done to analyze the potential of AI-based technology in radiology as well as in other applications of healthcare	Not applicable
13	[23]	Roles of regulators, accuracy, patient factors, physician factors	All the ethical principles that apply to the implementation of AI in healthcare emphasize ‘doing no harm’ to the patients	Components like mission training ethics shared ethics patient and physician-related ethics should be studied in detail so that universal regulations for the implementation of AI in medicine and ophthalmology are standardized	Not mentioned
14	[24]	Privacy, bias, transparency, interpretability, explainability, resource inequality, liability	AI technology can post difficult situations that can lead to misuse and unwelcoming response fields such as radiology	It is very important to understand the operation of this technology so that mindful decisions can be made in the application of the same in healthcare	Ethical codes should be developed for the use of AI highlighting the benefits as well as addressing the potential harms
15	[25]	Security, justice. Reliability	Artificial intelligence has the power to create remarkable breakthroughs in technology and advanced the diagnostic industry and lab medicine for the betterment of patients	The major challenges that AI poses are a complex mixture comprising of the privacy and reliability of these interventions in healthcare	Pathologists and laboratory professionals have the obligation to improve the ethical issues regarding the inclusion in healthcare proper validation and implementation should be done by the organizers as well as the stakeholders.
16	[26]	Autonomy, privacy and bias	The relationship between self-monitoring and ecological momentary assessment will it in the prevention of mental illness with the use of artificial intelligence	Although this method is efficacious challenges imposed are related to the autonomy, privacy and data security and the potential bias in these technologies	Not mentioned

**Table 4:** Timeline of studies included in this review.

Year	No of Studies = *n* (%)	Study ID
2014	1 (6.25%)	1
2018	2 (12.5%)	6, 12
2019	2 (12.5%)	2, 4
2020	5 (31.25%)	7, 8, 9, 14, 15
2021	4 (25%)	5, 10, 11, 13
2022	2 (12.5%)	3, 16

## Data Availability

The data that supports this study are available upon request from the corresponding author.
